# Depth-dependent mortality of reef corals following a severe bleaching event: implications for thermal refuges and population recovery

**DOI:** 10.12688/f1000research.2-187.v3

**Published:** 2014-02-19

**Authors:** Tom C. L. Bridge, Andrew S Hoey, Stuart J Campbell, Efin Muttaqin, Edi Rudi, Nur Fadli, Andrew H Baird

**Affiliations:** 1 ARC Centre of Excellence for Coral Reef Studies, James Cook University, Townsville, Queensland 4811, Australia; 2 Australian Institute of Marine Science, Townsville, Queensland 4810, Australia; 3 Wildlife Conservation Society, Indonesia Marine Program, Bogor, Indonesia; 4 Centre for Marine and Fisheries Studies, Syiah Kuala University, Banda Aceh, Indonesia

**Keywords:** Coral bleaching, climate change, Acropora, Aceh, Coral Triangle

## Abstract

Coral bleaching caused by rising sea temperature is a primary cause of coral reef degradation. However, bleaching patterns often show significant spatial variability, therefore identifying locations where local conditions may provide thermal refuges is a high conservation priority. Coral bleaching mortality often diminishes with increasing depth, but clear depth zonation of coral communities and putative limited overlap in species composition between deep and shallow reef habitats has led to the conclusion that deeper reef habitats will provide limited refuge from bleaching for most species. Here, we show that coral mortality following a severe bleaching event diminished sharply with depth. **Bleaching-induced mortality of *Acropora* was approximately 90% at 0-2m, 60% at 3-4 m, yet at 6-8m there was negligible mortality. Importantly, at least two-thirds of the shallow-water (2-3 m) *Acropora* assemblage had a depth range that straddled the transition from high to low mortality. Cold-water upwelling may have contributed to the lower mortality observed in all but the shallowest depths. Our results demonstrate that, in this instance, depth provided a refuge for individuals from a high proportion of species in this *Acropora*-dominated assemblage. The persistence of deeper populations may provide a critical source of propagules to assist recovery of adjacent shallow-water reefs.

## Introduction

Mass bleaching events causing extensive mortality of reef-building corals have become more frequent and widespread in recent decades and have affected almost all coral reef regions ^1– 3^. Coral bleaching is a generalised stress response resulting from numerous causes including sedimentation, freshwater exposure or disease ^4^; however, the most geographically extensive and severe events are correlated with sustained periods of elevated sea water temperatures and high light irradiance ^5^. The bleaching response is caused by the expulsion of a symbiotic dinoflagellate *Symbiodinium* that occur within the coral tissue and allow corals to harness energy from sunlight, thus providing a significant portion of the energy requirements. The sensitivity of this symbiosis to elevated sea temperature is well-documented ^5, 6^, suggesting that many coral species will be highly vulnerable to the effects of global warming ^7, 8^.

Despite this apparent sensitivity, reef corals have persisted through numerous large-magnitude and sometimes rapid changes in sea surface temperatures over the past 240 million years ^3, 9^. One mechanism by which a species can cope with changing local climate is to move to a more favourable area, and tropical reef corals have repeatedly shifted their distribution to higher latitudes in response to past climate warming ^10, 11^. Alternatively, populations may persist in microrefugia, defined as small areas of suitable habitat within regionally unfavourable environmental conditions ^12, 13^. Despite increasing recognition of their importance for conservation planning in terrestrial ecosystems ^14– 16^, microrefugia are less considered in the marine realm.

The severity of coral bleaching is often spatially heterogeneous due to both historical ^17, 18^ and environmental ^19– 21^ factors. Coral bleaching is caused by a synergistic effect between heat and light, and therefore microrefugia from bleaching are likely to occur in regions where oceanographic or atmospheric conditions reduce water temperatures or light irradiance relative to surrounding areas ^22^. Light irradiance declines with depth and ambient temperatures are often lower in deeper waters, therefore the incidence of bleaching and/or subsequent mortality is likely to be lower at greater water depths ^1, 5, 22^. Warm-water coral *bleaching* is occasionally reported to depths of 50 m, however, such observations are rarely followed up in order to estimate bleaching-induced *mortality*. Typically the incidence of bleaching is substantially lower at greater depths and in the few cases it has been measured, so is bleaching-induced mortality ^23– 25^. For example, mortality rates of corals at a depth of 6 m were only a third of those in 2 m across several turbid inshore reefs on the Great Barrier Reef (GBR) ^24^. A transition from high to low mortality with increasing depth was observed at numerous sites in the western Indian Ocean during 1998, the most severe and widespread bleaching event on record ^26^. This transition often occurred across a fairly sharp depth boundary at intermediate depths of 10–15 m ^26^, therefore species with depth ranges that straddle this transition from high to low bleaching mortality will have a refuge from bleaching in deeper water. However, most assessments of coral reefs consider only shallow habitats, and reductions in mortality with increasing depth may go unnoticed. Furthermore, recent studies of deep-water reefs have indicated that many corals may occur over a wider depth range than currently thought ^27, 28^.

In May–June 2010, a sustained increase in seawater temperatures in the Andaman and South China Seas resulted in extensive coral bleaching and caused high mortality of many coral species ^29^. Six weeks after the peak seawater temperatures, 45% of all corals and 94% of *Acropora* colonies were dead in shallow waters (1–2 m) around Pulau Weh, Sumatra, Indonesia ^29^. Here, we assess the effects of this severe thermal bleaching event at Pulau Weh over a depth gradient from 2–27 m to investigate 1) whether severe mortality of reef corals observed in shallow water (0–2 m) extended into deeper habitats; and 2) whether depth provided a refuge from bleaching mortality. We concentrate on the corals of the genus *Acropora* because they are the most diverse and abundant genus in the Indo-Pacific, and are important ecosystem engineers on most Indo-Pacific coral reefs. They are also often amongst the most susceptible taxa to bleaching-induced mortality, and bleaching events often result in shifts from *Acropora* – dominated communities towards communities dominated by more bleaching resistant taxa (e.g. *Porites* and the family Merulinidae) ^26, 30^. Change in *Acropora* cover before and after a bleaching event is therefore a useful indicator of bleaching severity.

## Materials and methods

**Figure S1.**
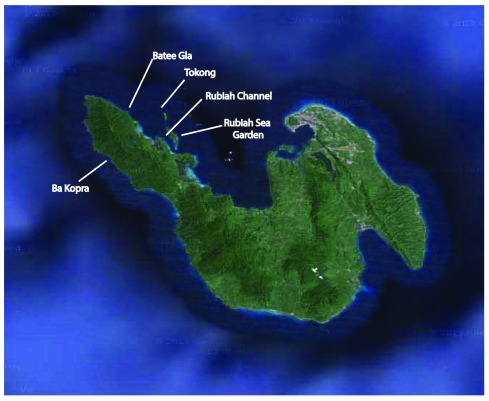
Map of Pulau Weh showing location of study sites.

Pulau Weh (5° 50’N, 95° 20’E) is located in the province of Aceh off the northwest coast of Sumatra, Indonesia. The region’s reefs have received little attention from scientists, but support similarly diverse coral communities to the rest of the Indo-Australian Archipelago ^31^. Northwest Sumatra was the epicentre of the December 2004 Indian Ocean tsunami, and although Pulau Weh’s coral communities were relatively unaffected by this event ^32^, they suffered substantial mortality in the 2010 Andaman Sea bleaching ^29^. To examine the influence of depth on bleaching mortality, we compared both total coral cover and *Acropora* cover collected before (November 2009 to February 2010) and after (July 2011) the bleaching event at three depths (0–2 m, 3–4 m and 6–8 m) at four sites on the northern and western sides of Pulau Weh (Batee Gla, Ba Kopra, Rubiah Sea Garden, Rubiah Channel – Figure S1). Coral cover was estimated along 6–10 replicate 10 m line intercept transects, which were haphazardly placed at 0–2 m, and 3–6 replicate 50 m point intercept transects at 3–4 and 6–8 m (see Data File). Any live hard coral (i.e. scleractinian or hydrozoan coral) underlying each survey point was recorded to genus level. Changes in total live coral cover and *Acropora* cover between 2009 and 2011 were compared using two-factor ANOVA’s. Assumptions of the ANOVA’s were examined using residual analysis and no transformation was necessary. The analyses were based on the proportion of total coral or *Acropora* cover per 50 m transect.

To determine the proportion of the *Acropora* assemblage afforded a depth refuge from this bleaching event, we conducted species-level surveys of *Acropora* assemblages in 0 to 2 m and then at 5 m intervals from 3–27 m in February 2012 at five sites on the northern and western sides of Pulau Weh (Batee Gla, Ba Kopra, Rubiah Sea Garden, Rubiah Channel and Tokong - Figure S1. Sites were chosen based on their bathymetry profiles, with accessible deep sites only present on the steeply-sloping, ocean-facing northern and western coasts. Data were collected at 5 m depth intervals using replicate 10-minute timed swims, where the species identity of every living *Acropora* colony was recorded. Post-bleaching surveys were compared to shallow-water (0–2 m) surveys conducted in November 2009 before the bleaching event using 40 min timed swims ^31^ at these same sites. Corals were identified using taxonomic references provided in “Staghorn Corals of the World” by Wallace CC and “Corals of the World”, by Veron JEN ^33, 34^. Analysis of Similarities (ANOSIM), a multivariate approximation of ANOVA ^35^, was performed on a square root-transformed Bray-Curtis similarity matrix to determine any significant difference in the *Acropora* assemblage among sites.

Total hard coral cover and Acropora cover for each transect pre-bleaching (2009) and post-bleaching (2011) “Bleaching” denotes whether the survey was pre-bleaching (2009) or post-bleaching (2011). “Depth” is the depth of the survey in metres, “Transect” is the transect number, “Acropora” denotes the % cover of Acropora in each transect, and “Total Hard Coral” is total coral cover for each transect.

## Results and discussion

**Figure 1.**
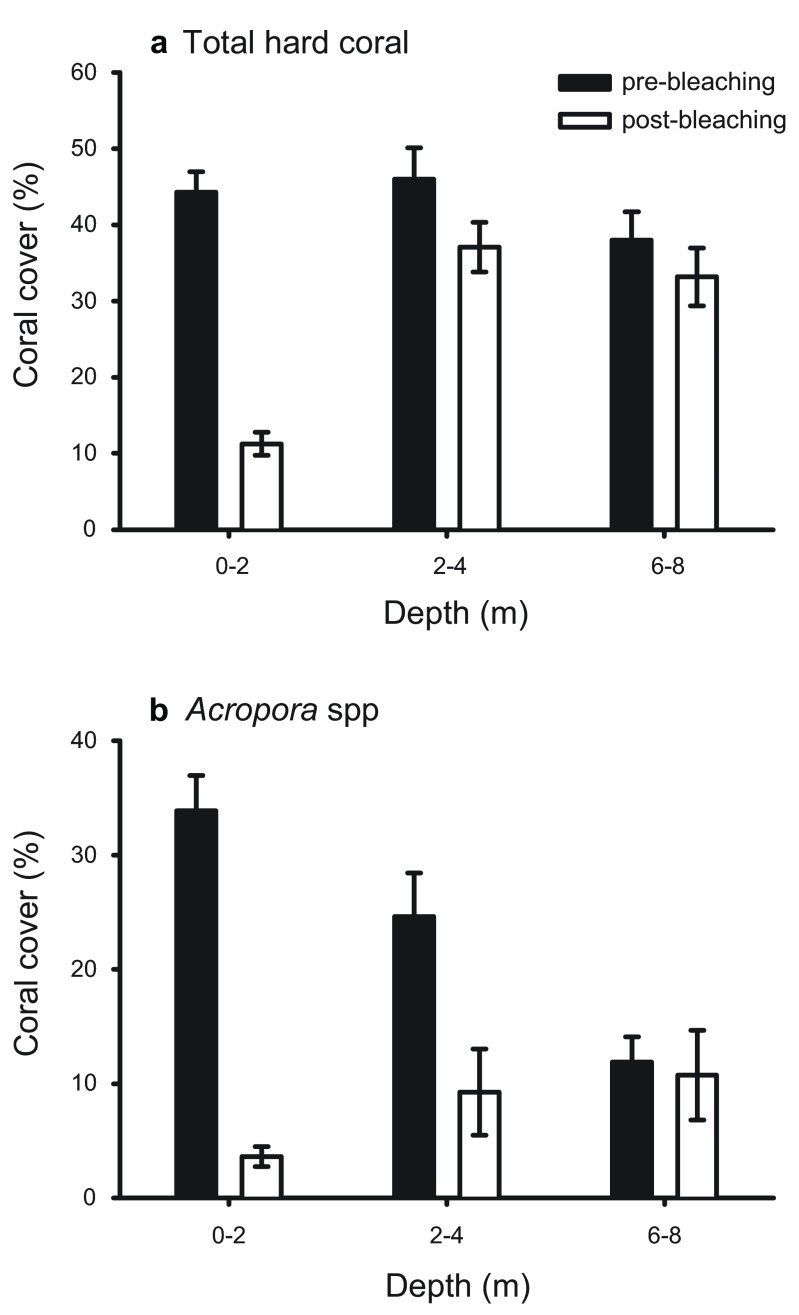
Change in live coral cover on Pulau Weh following the 2010 bleaching event at depths of 0–2, 2–4, and 6–8 metres. ( **a**) total live coral cover; and ( **b**) live *Acropora* cover.

A total of 40 *Acropora* species were observed during the study, confirming the high diversity previously reported on Acehnese reefs ^31^. ANOSIM revealed no significant difference in assemblage structure among sites, which were therefore pooled for further analysis. Bleaching mortality was very high in the shallows, however, mortality diminished rapidly with increasing depth ( Figure 1). Total coral cover declined by 75% at 0–2 m but only 20% at 3–4 m, while there was no significant change at 6–8 m ( Figure 1a; 2-way ANOVA depth by year interaction; F _2,123_ = 21.2, *p* < 0.001). The decline in mortality was even more pronounced in the *Acropora*, with cover declining by approximately 90% at 0–2 m and 60% at 3–4 m, with no change detected at 6–8 m ( Figure 1b; 2-way ANOVA depth x year; F _2,123_ = 17.9, *p* < 0.001).

**Figure 2.**
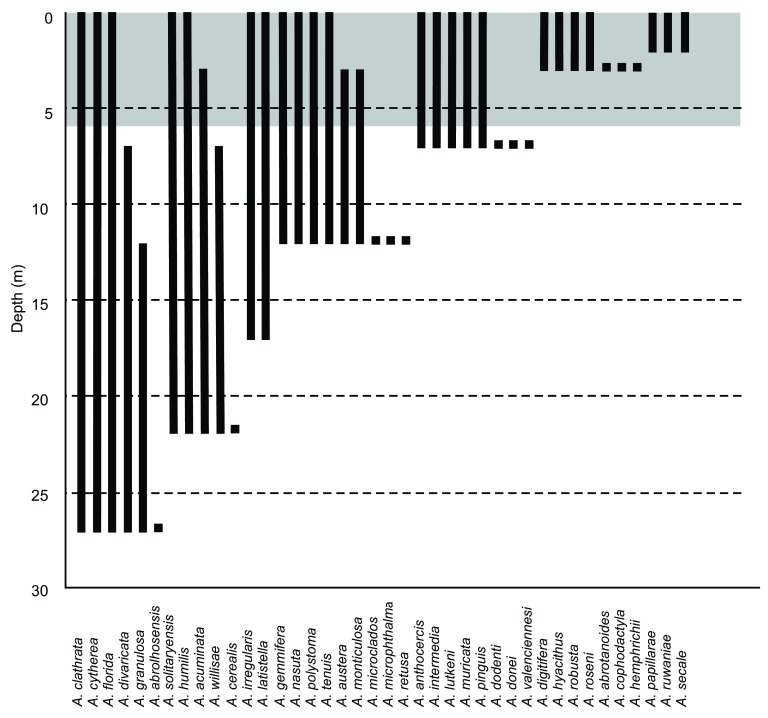
Depth distribution of 40 *Acropora* species observed at Pulau Weh following the bleaching event. Shaded (grey) panel indicates the depth range where bleaching mortality was high. Of the 29 species occurring in 0–7 m depth, 19 (66%) also occurred below 7 m.

A high proportion of this diverse *Acropora* assemblage was afforded a refuge from bleaching mortality in deeper water. Of the 29 *Acropora* species occurring in shallow waters < 7 m, 19 (66%) also occurred below the approximate depth of transition from high to low mortality ( Figure 2). However, the refuge effect would be diminished if mortality had reached into deeper waters. If, for example, the transition between high and low bleaching mortality had occurred at 12 m, 14 (48%) of the species affected would have had a refuge in depth. Similarly, if bleaching mortality extended to 22 m, only 6 species from the shallow assemblage (21%) would have had colonies persisting below the transition depth.

**Figure 3.**
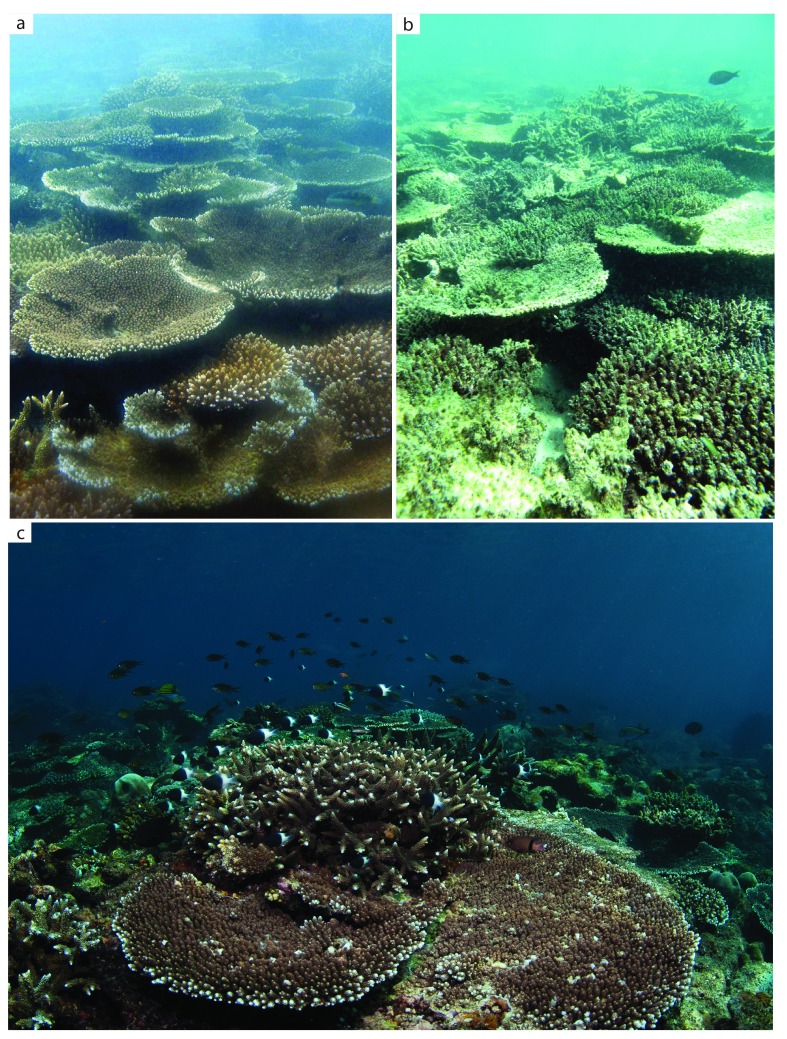
*Acropora*-dominated communities at Pulau Weh. ( **a**) Reef crest at 2 m depth prior to bleaching (16 November 2009); ( **b**) the same reef crest six weeks after the peak of bleaching (26 July 2010); ( **c**) upper reef slope community at 6 m depth largely unaffected by the bleaching event, 25 February 2012.

Doubts regarding the potential significance of depth as a refuge for corals from warm-water bleaching have previously been raised because (1) bleaching has been observed in the deeper areas of reefs, (2) there is limited overlap of species between deep and shallow reef areas, and (3) genetic partitioning within species among depths suggests that deeper population cannot provide an effective source of recruits for shallow populations ^36, 37^. Firstly, while *bleaching* often extends to the lower depth limits of some shallow water species, both bleaching frequency and, most importantly, *mortality*, is often strongly depth dependent ( Figure 3) ^24, 26, 38^. Indeed, a transition from high to low mortality occurred at depths of ≤ 15 m ~50% of sites surveyed in the Indian Ocean in 1998 ^26– see Table 1^). Secondly, our results indicate that even with a pronounced depth zonation in the *Acropora* assemblage, two-thirds of species occurring in shallow depths had a depth range that straddled the transition in bleaching mortality. The depth zonation of coral assemblages is one of the most consistent and predictable patterns in nature ^39, 40^ and therefore our results are not an anomaly. Thirdly, the genetic divergence between populations above and below the transition in mortality at between 4 and 8 m is unlikely to be sufficient to prevent larval migration in either direction. For example, larvae of the coral *Seriatopota hystrix* migrate among sub-populations over a 30 m depth range ^41^. Furthermore, connectivity modelling in two Caribbean coral species indicates demographically significant larval subsidy from deep to shallow reef habitats over a much greater depth range (5–40 m) even when deep-water fertilisation rates and post-settlement survival are greatly reduced ^42^.

Our results, and those of previous studies ^24, 26, 38^, indicate that bleaching mortality can vary considerably over a small depth range. Consequently, surveys conducted at a single depth may greatly misrepresent the proportion of coral populations killed by coral bleaching ^29^. For example, long-term, large-scale monitoring of coral cover on reef slopes (6–9 m depth) on the GBR suggests that bleaching has been a comparatively minor source of coral mortality over the last few decades ^43, 44^, despite two mass bleaching events in 1998 and 2002 ^45^. However, in the 1998 bleaching event on the inshore GBR, bleaching mortality was on average 3-times higher at 2–4 m when compared to 5–8 m ^24^. Clearly, ecosystem assessments considering only a single depth may provide a biased view of the relative importance of the many different agents of coral mortality, and should therefore be conducted over a range of depths to accurately assess the relative importance of multiple stressors.

Identifying areas or conditions that consistently provide refuges for corals from thermal stress is critically important for coral reef conservation under future climate change. In 1998, lower mortality and a shallower transition depth was often associated with sites that experienced episodic upwelling of cold water ^26, 46, 47^. Although environmental data are not available from Pulau Weh, pulses of cold water were regularly experienced during data collection, and rapid upwelling-driven temperature plunges of up to 10°C are recorded from the west coast of the nearby Similan Islands ^48^. Interestingly, Acehnese reefs appeared unaffected by the 1998 bleaching event ^29^, despite the coral bleaching extending across virtually the entire Indian Ocean from east Africa and north-western Australia ^26, 49, 50^. These cold-water upwelling events may explain the lack of mortality in 1998 and the shallow transition depth during 2010 despite very high sea surface temperatures. Depth-dependent mortality was most evident at Ba Kopra, on the western side of Pulau Weh ( Figure S1), supporting the hypothesis that these upwelling events may create small-scale refugia from thermal anomalies. If so, this region may provide a consistent refuge for many corals against rising sea temperatures. In summary, our results show that coral bleaching mortality can diminish rapidly even where shallow-water corals experience severe mortality, and modest depths can provide a refuge for a significant proportion of coral species. Identifying sites where oceanographic conditions reduce the effects of thermal anomalies should be a priority for coral reef conservation.
