# Supplementary material for: Depth-dependent mortality of reef corals following a severe bleaching event: implications for thermal refuges and population recovery
Source: F1000Res. 2014 Feb 19;2:187. Originally published 2013 Sep 16. [Version 3] doi: 10.12688/f1000research.2-187.v3 (PMC3938179; doi:10.12688/f1000research.2-187.v3)
Supplement: Peer review history

# Peer review: Referee response for version 3

**DOI:** 10.5256/f1000research.3868.r4852

**Published:** 2014-05-22

**Authors:** Bert Hoeksema

**Affiliations:** 1 Netherlands Centre for Biodiversity Naturalis, Leiden, Netherlands

I have read this submission. I believe that I have an appropriate level of expertise to confirm that it is of an acceptable scientific standard.

# Peer review: Referee response for version 2

**DOI:** 10.5256/f1000research.2830.r3294

**Published:** 2014-01-24

**Authors:** Bert Hoeksema

**Affiliations:** 1 Netherlands Centre for Biodiversity Naturalis, Leiden, Netherlands

This article presents information on the depth-related occurrence of bleaching among 40 *Acropora *species around Pulau Weh, off North Sumatra. The article is providing important data. It is written in a concise way but I suggest that the authors add a bit more nuance to their argumentation.

**Title**: The authors could be more specific regarding mentioning the selection of coral species in the title. The contents of the paper is not about a representative selection of ”reef corals” mainly about staghorn corals, *Acropora *species, which according to the authors “are also often amongst the most susceptible taxa to bleaching-induced mortality”.

**Figure 2**: The caption is confusing. It mentions the bathymetrical distribution of 41 *Acropora* species, and the depth range where mortality as a result of bleaching was reported to be high. The main text mentions 40 *Acropora *species. Does bleaching mortality in 0-2 m refer to all corals or just *Acropora* corals, because it seems that (almost) no *Acropora* corals were found here. The authors should explain that this figure represents a mix of data from 2009-2011 (0-8 m: i.e. 0-2, 3-4, 6-8 m) and from 2012 (2-27 m: i.e. 2, 7, 12, 17, 22, 27 m).

**Results and discussion: **The sentence “Doubts regarding the potential significance of depth as a refuge for corals from warm-water bleaching have previously been raised because…” seems to overlook various papers that do agree with depth-related coral bleaching (e.g. Hoeksema 1991, Marshall & Baird 2000, Craig *et al.* 2001, Furby *et al.* 2013).

The sentences “Our results indicate that bleaching mortality can vary considerably over a small depth range. Consequently, surveys conducted only in shallow waters may greatly overestimate the proportion of coral populations killed by coral bleaching” overlook the fact that some coral species show less bleaching on sun-exposed reef flats, suggesting that they are pre-adapted to high water temperatures (e.g. Hoeksema 1991, Ware *et al.* 1996, Craig *et al.* 2001, Hoeksema & Matthews 2011).

Since *Acropora* corals were identified at species level in 2012, is there a way to tell whether the species showed interspecific differences in bleaching (see Hoeksema 1991, Craig *et al.* 2001, Hoeksema & Matthews 2011)?

I have read this submission. I believe that I have an appropriate level of expertise to confirm that it is of an acceptable scientific standard.

# Response

**Published:** 2014-02-21

**Authors:** Tom Bridge

**Affiliations:** James Cook University, Australia

- *“The authors could be more specific regarding mentioning the selection of coral species in the title. The contents of the paper is not about a representative selection of ”reef corals” mainly about staghorn corals, Acropora species, which according to the authors “are also often amongst the most susceptible taxa to bleaching-induced mortality””*
  We do assess depth-dependent mortality for all corals, but focus on depth distributions of Acropora because they were the dominant group at these sites. Therefore, we believe the title accurately reflects the nature of the research.
- **“Does bleaching mortality in 0-2 m refer to all corals or just Acropora corals, because it seems that (almost) no Acropora corals were found here. The authors should explain that this figure represents a mix of data from 2009-2011 (0-8 m: i.e. 0-2, 3-4, 6-8 m) and from 2012 (2-27 m: i.e. 2, 7, 12, 17, 22, 27 m).” **
  All of the data presented in Figure 2 is from 2012 (post-bleaching), as stated in the Methods. In Figure 2, the bars on the species encountered in the shallowest depth range (0-2 m) have been extended to 0 m to indicate that they could have occurred anywhere from 0-2 m depth.
- *“The sentence “Doubts regarding the potential significance of depth as a refuge for corals from warm-water bleaching have previously been raised because…” seems to overlook various papers that do agree with depth-related coral bleaching” *
  Our discussion states that various authors have discounted the depth refuge effect by several mechanisms. However, we go on to cite examples ( Marshall & Baird, 2000; Sheppard & Obura, 2005) of studies that report depth-dependent coral bleaching, demonstrating that the depth-dependent bleaching observed in this study is not an isolated case. We have added a citation to Hoeksema (1991) as a further example of depth-dependent coral bleaching.
- *“The sentences “Our results indicate that bleaching mortality can vary considerably over a small depth range. Consequently, surveys conducted only in shallow waters may greatly overestimate the proportion of coral populations killed by coral bleaching” overlook the fact that some coral species show less bleaching on sun-exposed reef flats, suggesting that they are pre-adapted to high water temperatures”* 
  To clarify, we have altered the text to state that “Our results, and those of previous, indicate that bleaching mortality can vary considerably over a small depth range. Consequently, surveys conducted at a single depth may greatly misrepresent the proportion of coral populations killed by bleaching”.
- *“Since Acropora corals were identified at species level in 2012, is there a way to tell whether the species showed interspecific differences in bleaching?” *
  We could test for interspecific differences in mortality among some of the more abundant species; however, this is beyond the scope of the current manuscript, which focuses on depth-dependent mortality.

# Peer review: Referee response for version 2

**DOI:** 10.5256/f1000research.2830.r2275

**Published:** 2013-12-05

**Authors:** John Rooney

**Affiliations:** 1 Coral Reef Ecosystem Division, National Oceanic and Atmospheric Administration, Washington, DC, USA

The authors report that coral bleaching at their study site declined dramatically with increasing depth and they hypothesize that the mitigation of bleaching was caused by localized decreases in temperature caused by cold water upwelling. Although they are continuing their research at the site, including monitoring temperature, at the time they wrote this article no data were available to discuss the magnitude and temporal characteristics of temperature differences between heavily and lightly or unbleached sites. However, the manuscript would be improved with a brief discussion of the magnitude and duration of temperature differences that have been reported in the scientific literature (e.g. Hoegh-Guldberg, 1999) that have been found to induce bleaching.

I have read this submission. I believe that I have an appropriate level of expertise to confirm that it is of an acceptable scientific standard.

# Response

**Published:** 2014-02-21

**Authors:** Tom Bridge

**Affiliations:** James Cook University, Australia

In the literature, there is a large range of temperatures reported as thresholds to induce bleaching, and considerable variation in these thresholds due to geographic location, composition of the assemblage, thermal history etc. It is certainly an interesting question, but we maintain that without any data any attempt to identify thresholds in this case would be overly speculative. As such, we do not believe this is the correct venue to incorporate such discussion.

# Peer review: Referee response for version 1

**DOI:** 10.5256/f1000research.2162.r1825

**Published:** 2013-10-02

**Authors:** John Rooney

**Affiliations:** 1 Coral Reef Ecosystem Division, National Oceanic and Atmospheric Administration, Washington, DC, USA

This paper makes a valuable contribution, highlighting the marked differences in bleaching-induced coral mortality associated with changes in depth of just a few meters. It highlights the need to conduct surveys over a range of depths to characterize bleaching events and, in particular, to identify “microrefugia” - sites where oceanographic conditions reduce the effects of thermal anomalies - as a priority for coral reef conservation. 

 In their introduction the authors mention the importance of both irradiance and temperature, but no further mention of irradiance's possible role in the observed coral bleaching and mortality patterns is made. Additionally, the author’s state that “pulses of cold water were regularly experienced during data collection” as evidence that upwelling of cold water was the mechanism responsible for the reduced bleaching-induced coral mortality at their deeper survey sites. Although it may not be possible to reconstruct temperature differences during the 2010 bleaching event at their study sites, even temperature records from the different survey depths on the Acehnese reefs well after the event may provide some insight into the possible magnitude of temperature differences that were sufficient to reduce coral mortality.  Some discussion of the specific parameters that might distinguish microrefugia, e.g. the frequency and magnitude of differences in temperature or irradiance relative to surrounding waters, would greatly enhance the paper’s utility, and provide an important addition to further work on this topic.

I have read this submission. I believe that I have an appropriate level of expertise to confirm that it is of an acceptable scientific standard.

# Response

**Published:** 2013-10-27

**Authors:** Tom Bridge

**Affiliations:** James Cook University, Australia

Unfortunately, no data are currently available to quantify the local oceanographic patterns at this time, and to address this question without such data would be overly speculative. However, we plan to collect temperature data at Pulau Weh as part of ongoing monitoring of reefs in the region.

# Peer review: Referee response for version 1

**DOI:** 10.5256/f1000research.2162.r1823

**Published:** 2013-09-20

**Authors:** Tyler Smith

**Affiliations:** 1 Center for Marine and Environmental Studies, University of the Virgin Islands, St Thomas, Virgin Islands, U.S.

The article is of great interest considering the impact of thermal stress on coral reefs globally, and the pressing need to identify refugia that might support coral diversity in a warming ocean. The article is basic, in that it only attempts to directly answer two questions: (1) did acroporid corals do better at deeper depths (8m) versus shallower depths (2 and 4m), and (2) is there a significant proportion of acroporid diversity that would be protected by the identified depth refuge in 2010 (i.e., how many acorporid species have a sufficiently wide depth range). I believe that they answer these questions well, but I would have liked to see more thorough investigation of the patterns in the data and I think there are some anomalous parts of the data that I cannot explain. For example, while most sites followed a pattern with increasing bleaching mortality at shallower depths, plotting of the data provided in table 1 shows that one site, Ba Kopra at 4m, showed no change or even an increase in total and acroporid cover, respectively. The authors never discuss this site to site variability, which might be important for "Identifying sites where oceanographic conditions reduce the effects of thermal anomalies should be a priority for coral reef conservation". 

As for anomalous parts of the data, I can't understand why the absolute cover change of acroporid corals seems to surpass that of total coral cover at some sites. For example, at Rubiah Channel the acroporid cover drops an absolute amount of 38.1%, by my calculation. That is a simple calculation of final cover - initial cover, not standardized to the initial coral cover (which would be relative cover change). Therefore, the total absolute coral cover change has to be at least 38.1%, yet it is only 23.6%. This is not that the total cover is really the total cover excluding acropora, since the prior to bleaching acropora + total cover = 102%. Can the authors explain this? 

In the methods it would also be necessary to know if the pre- and post-bleaching transects are the same (i.e., fixed permanent transects) or whether they are randomly placed. In either case, but particularly for the latter, it is also necessary to know the method by which the placement of the transect was determined at a site. How are we to know potential biases in the pre- and post-assessments without this knowledge. There is also no information given on the exact location of monitoring sites, which is important for replication of the study. Perhaps coordinates and a map would be appropriate. 

One further addition that would be nice, though an addition to the two primary questions of the manuscript, is what is the relative importance of the depth refuge to the *in situ* shallow coral survival? They mention "recovery" in the title, but this isn't really addressed much in the manuscript, and improving the discussion of potential recovery process would help on that point. I.e., what absolute amount of cover for each species survived deep, and could contribute to shallow water recovery via larval recruitment, versus the amount that survived shallow and could contribute to direct asexual recovery and larval recruitment? Also, any speculation on the relative importance of the processes in recovery (deep to shallow larval recruitment versus shallow to shallow larval and asexual recruitment) would be a good addition and set up future research.

I have a feeling all these questions are easily answerable and that the conclusions are justified, and I think the manuscript is an important addition to a rather sparse body of knowledge concerning reef refuges and refugia.

I have read this submission. I believe that I have an appropriate level of expertise to confirm that it is of an acceptable scientific standard, however I have significant reservations, as outlined above.

# Response

**Published:** 2013-10-27

**Authors:** Tom Bridge

**Affiliations:** James Cook University, Australia

Our response to the issues raised by the reviewer are summarised below:

- We acknowledge some variability among sites in the depth of transition from high to low mortality, but the overall trend of decreasing mortality with increasing depth is consistent among sites.
- The reviewer noted that the ‘absolute cover change of Acroporid corals seems to surpass that of total coral cover’, and we note three possible reasons for this observation.  Firstly, such variations are commonly observed on coral reefs after a disturbance event such as coral bleaching due to ‘canopy effects’, whereby removal of canopy-forming taxa (in this case *Acropora*) can cause apparent increases in taxa which were concealed underneath the canopy. Canopy effects have been well documented in the coral reef literature (e.g. Goatley CHR & Bellwood DR, 2011). In Pulau Weh, the increases in encrusting/massive *Porites* and *Faviids *suggest canopy effects were a likely cause of these observations. Secondly, surviving corals will have grown from 2009 to 2011. Thirdly, transects were haphazardly placed and therefore some variability within a site is possible from year to year.
- We have included a map of study sites, and also stated in the text that transects were haphazardly placed within a site.
- Coral cover data from 2009 to 2011 was collected only to genus level, therefore it was not possible to address species-specific changes in abundance before and after the bleaching.
